# Glamour of technology

**DOI:** 10.4103/0971-6203.66759

**Published:** 2010

**Authors:** Bhudatt R. Paliwal

**Affiliations:** Department of Human Oncology, University of Wisconsin-Madison, 600 Highland Avenue, K4/B47, Madison, WI 53792, USA. E-mail: paliwal@humonc.wisc.edu

Practitioners of Medical Physics are among the major developers, promoters, and users of technology in the cancer field. A large number of hardware and software tools have formed the backbone of imaging-based early detection and treatment of myriad of malignancies. These developments have produced a plethora of products and have made significant contributions to the likelihood of cure and quality of life of cancer patients. Further progress in developing newer technologies and refining older ones requires an audience of peers with whom these experiences can be shared and from whom constructive critiques can be received. Rising above the multiple peer-reviewed journals facilitating this peer review discussion, *Journal of Medical Physics* continues to provide service to the international community of physicists and physicians.

The rapidity of advances has in large part been facilitated by the emergence of digital technology. Its efficiencies in collecting data that can be aggregated, analyzed, redistributed, and shared has enabled the generation of new insight and experimentation of novel solutions to ongoing diagnostic and therapeutic challenges. There are abundant examples of these applications in management, radiation detection, radiologic imaging, and radiation oncology[1–5] as well as in other oncologic and nononcologic disciplines. These technological developments appeal to physicists, physicians, and patients as faster, smaller, easier, and less invasive tools and procedures. The future trend is toward more personalized care. We are going to see technology that will surprise us and change the way we practice and provide services to patients.

However, the rapidity of dissemination of new technology comes with multiple costs, such as the overutilization[6,7] of new technology by undertrained personnel. A recent analysis (source: New York State Department of Health, *New York Times* analysis) describes 621 errors from January 2001 to January 2009 in the state of New York. These errors included missing all or part of the treatment, delivering the wrong dose, and even treating a patient with another patient’s prescription. A large number of these errors were technology driven, such as digital data transfer, or the inability of humans to properly respond to technological complexities, such as the malfunction of computer software.

In addition, even though radiation therapy may provide a greater financial value compared to newer systemic therapies, the rapidly increasing overutilization of high-end technology is contributing to higher cost and suffering.[8,9] There have been spectacular advances in achieving higher resolution, rapid volumetric reconstruction, and auto-segmentation techniques. However, the rapid proliferation of these technologies has resulted in the performance of questionably unnecessary procedures at added radiation exposures and financial cost to the society. There is no doubt that new technology benefits some patients, but it also promotes waste and duplicative applications.

We also need to assess the potential for long-term sequelae, such as carcinogenesis.[10–12] Long-term effects of low levels of irradiation remain underappreciated. Cancer risks at high doses are well-known from epidemiologic studies of the Japanese survivors. Absent further data, risks at low doses must be extrapolated from the high-dose data.

It is important to ask if more of the same is going to produce a better treatment outcome? Will our ability to calculate instantaneously, and measure and deliver radiation to a greater and greater precision and accuracy provide greater cure rates? The discipline of radiation therapy, for example is changing. In some malignancies, such as head and neck and gastrointestinal sites, the role of radiation has solidified while in others either it has remained stable or has declined (pediatrics and lymphoma). In metastatic diseases, it is mostly used in conjunction with chemotherapy where greater emphasis is on systemic disease. Proton beams are also in use for delivering higher and conformal dose to the tumor in the hope of achieving an increased tumor control probability. Some clinical gains with protons have been realized in the treatment of uveal melanomas and sarcomas of the base of skull.[13] If we look at a bigger picture, it could be argued that it is not any individual technology. Instead it is the combined modality approach that has contributed mostly to some improvements in patient survival. Even though radiation therapy provides a relatively greater value compared to systemic therapies, its high-end technology and widespread overutilization are contributing to a higher cost. Similar considerations apply to noninvasive multimodality imaging procedures. Dose escalation studies, driven by the hypothesis that dose nonuniformity within the planning target volume may lead to an increase in local control, have boosted the interest in more precise morphological and biological delineation of the target volume.[14] There have been spectacular advances in achieving higher resolution, rapid volumetric reconstruction, and auto-segmentation techniques. Rapid proliferation has resulted in unnecessary procedures at added radiation exposures and financial cost to the society. Recently, there has been an effort to integrate MR guidance with radiation delivery machines. Several MR-guided radiation treatment systems are currently underdevelopment.[15–17] New technology is a boon to some patients, but it also promotes overutilization[18] of technology-driven applications.

As writers and readings of an academic peer-reviewed medical physics journal, our responsibility toward our patients and toward the medical community is to “wipe away the shine” of new technology and question the value of technological advances. Will more of the same produce a better treatment outcome? Will our ability to calculate instantaneously, and measure and deliver radiation to a greater precision and accuracy provide higher cure rates? Will we be able to implement more rigorous safeguards and better train the handlers of new technology?

Our medical community needs to find a balance between promoting new developments and emphasizing rigorous standards for evidence of clinical benefit. A healthy approach would be to self-regulate by establishing stricter guidelines and protocols for the implementation of new technology. The absence of an internally established discipline could invite regulatory intervention, usually detrimental to growth and limiting benefits to potentially deserving patients.
